# Al_2_O_3_-Cu-Ni Composites Manufactured via Uniaxial Pressing: Microstructure, Magnetic, and Mechanical Properties

**DOI:** 10.3390/ma15051848

**Published:** 2022-03-01

**Authors:** Paulina Piotrkiewicz, Justyna Zygmuntowicz, Marcin Wachowski, Konrad Cymerman, Waldemar Kaszuwara, Anna Więcław Midor

**Affiliations:** 1 Faculty of Materials Science and Engineering, Warsaw University of Technology, 141 Woloska St., 02-507 Warsaw, Poland; paulina.piotrkiewicz.dokt@pw.edu.pl (P.P.); konrad.cymerman.dokt@pw.edu.pl (K.C.); waldemar.kaszuwara@pw.edu.pl (W.K.); 2 Faculty of Mechanical Engineering, Military University of Technology, 2 gen. S. Kaliskiego St., 00-908 Warsaw, Poland; marcin.wachowski@wat.edu.pl; 3 Faculty of Chemistry, Warsaw University of Technology, 3 Noakowskiego St., 00-664 Warsaw, Poland; awieclaw@ch.pw.edu.pl

**Keywords:** Al_2_O_3_-Cu-Ni, uniaxial pressing, composites

## Abstract

This study’s main goal was to obtain and characterize Al_2_O_3_-Cu-Ni composites with different metallic phase content. The study analyzed the three series of samples differing in the metallic phase 5, 10, 15 vol.% volume contents. An identical volume share of the metallic components in the metallic phase was used. Ceramic–metal composites were formed using uniaxial pressing and sintered at a temperature of 1400 °C. The microstructural investigation of the Al_2_O_3_-Cu-Ni composite and its properties involved scanning electron microscopes observations and X-ray diffraction. The size of the metallic phase in the ceramic matrix was performed using a stereological analysis. Microhardness analysis with fracture toughness measures was applied to estimate the mechanical properties of the prepared materials. Additionally, magnetic measurements were carried out, and the saturation magnetization was determined on the obtained magnetic hysteresis loops. The prepared samples, regardless of the content of the metallic phase in each series, were characterized by a density exceeding 95% of the theoretical density. The magnetic measurements exhibited that the fabricated composites had ferromagnetic properties due to nickel and nickel-rich phases. The hardness of the samples containing 5, 10, 15 vol.% metallic phases decreased with an increase in the metallic phase content, equal to 17.60 ± 0.96 GPa, 15.40 ± 0.81 GPa, 12.6 ± 0.36 GPa, respectively.

## 1. Introduction

The ceramic–metal composites are new, interesting materials whose properties are the results of combining the properties of ceramic and metallic phases. In accordance with the structure, we can distinguish between composites with a ceramic and a metallic matrix. Depending on the ceramic matrix material applied and the type of metallic phase, ceramic–metal composites are widely used for industrial applications such as aerospace [1,2,3,4,5,6]. The composites with the expected properties and microstructure may be obtained by using one of many different methods of fabrication such as powder metallurgy methods [7,8], in situ methods [9], methods based on the metal infiltration [10], electrophoretic deposition [11], or combination of isostatic pressing and sintering [12]. Manufacturing methods that use slurries are used as well [13]. The choice of forming method affects the type of structure of the final material. Depending on the fabrication method, it is possible to obtain a homogeneous distribution of reinforcement, gradient structures, phase percolation, and layered composites.

Interesting and perspective examples of a ceramic–metal composite are Al_2_O_3_-Cu [14] and Al_2_O_3_-Cu-Ni composites [15], which have applications in the electronics, microelectronics, energy, and automotive industry. Due to the high thermal and electrical conductivity and high corrosion resistance, copper is used as a metallic phase in both composites. The main problem of obtaining alumina–copper composites is a weak connection of copper with ceramic components due to poor adhesion and the wettability between the solid and liquid phases during sintering. Another problem is a tendency to flow out the liquid copper from the sample during sintering. Therefore, research on the fabrication of composites that include copper as a metallic filler is still ongoing. The published research results showed that adding additional metallic components such as Cr, Mo, W, or Ni prevents leakage of liquid copper during sintering [16,17,18,19,20,21].

In this study, copper was partially replaced by nickel. Before sintering mixture of Ni and Cu powders was added to the composite as reinforcement. The sintering temperature of the Al_2_O_3_ matrix composites is about 1400 °C, and due to the different melting points of the phases (melting point for Ni = 1455 °C, melting point for Cu = 1085 °C), lower for the metals than ceramic, sintering will be performed in the liquid phase. During the sintering process, a liquid phase appears after the exceeded melting point of Cu (1085 °C), interacting with the nickel particles. Based on the Cu-Ni phase equilibrium system, it can be concluded that the Ni particles can completely dissolve in the liquid copper at the sintering temperature, and the entire metallic phase will form a liquid solution [22]. In practice, the mutual arrangement of the particles of both powders will affect the degree of conversion of both metallic components.

The addition of Ni powder may be beneficial because of how nickel facilitates the spread of liquid copper. It is due to liquid copper wets nickel very well, and the contact angle of nickel with copper is relatively low, reaching a value of 15–20° [23,24]. Nickel dissolving in copper can improve the wettability between the solution and the alumina matrix. Unfortunately, there are no data in the literature about the influence of Ni content on Al_2_O_3_ wettability by a Cu-Ni solution. It can be expected that the final sintering effect will be significantly dependent on the possibility of a reaction between the Cu and Ni particles. It depends on the initial concentration of the powders and the parameters of the sintering process: temperature, time, and pressure.

In this study, Al_2_O_3_-Cu-Ni composites with different metallic phase content are characterized. Uniaxial pressing was chosen as the fabrication method. It is one of the simplest powder metallurgy methods to produce metal–ceramic composites. This method makes it possible to produce samples from powder masses with a moisture content below 6% [25,26]. In this technique, two types of uniaxial pressing—one side and two sides—are possible [25,26]. This allows applying pressure only from above or simultaneously from two directions: above and below the sample. The advantage of the uniaxial pressing method is the economic aspect related to high efficiency and a small amount of waste. An additional factor determining the choice of this forming technique is the shape’s dimensional accuracy of the obtained shapes [25,26].

The main objective of this research was to obtain homogeneously Al_2_O_3_-Cu-Ni composites by uniaxial pressing, which were sintered at a temperature of 1400 °C. Three series of composites were produced. The obtained series was characterized by differences in the content of the metallic phase: series I—5 vol.% of the metallic phase, series II—10 vol.% of metallic phase, and series III—15 vol.% of metallic phase. In the prepared composites, the ratio of Cu to Ni was 1:1 in each sample (series I—Cu:Ni—2.5 vol.%:2.5 vol.%; series II—Cu:Ni—5 vol.%:5 vol.%; series III—Cu:Ni—7.5 vol.%:7.5 vol.%).

The resulting materials were evaluated in their microstructure, physical, magnetic, and mechanical (hardness, fracture toughness) properties. In addition, a stereological analysis was performed to determine the size of the metallic phase in the ceramic matrix. The phase composition of the manufactured samples was also tested.

The investigation presented in the manuscript allows obtaining knowledge about the correlation between parameters of the fabrication process and selected properties of the specimens from the Al_2_O_3_-Cu-Ni system. The research allowed gaining knowledge about the effect of changes in the metallic phase content in composites from the Al_2_O_3_-Cu-Ni system. The accomplishment of the suggested study methodology provided fundamental knowledge about the magnetic and mechanical properties of Al_2_O_3_-Cu-Ni composites obtained via uniaxial pressing. Moreover, obtained results will allow for a fully conscious selection of components of specimens based on the characterization of obtained materials.

## 2. Materials and Methods

### 2.1. Materials

The following commercially available powders were used for composite samples: Ceramic powder Al_2_O_3_ TM-DAR (Taimei Chemicals Co., Ltd., Tokyo, Japan) and Cu and Ni metallic powders (Createc, Stalowa Wola, Poland). The properties of the base materials are presented in Table 1. The parameters in Table 1 were summarized based on manufacturer data.

### 2.2. Preparation of Samples

**Figure 1:**
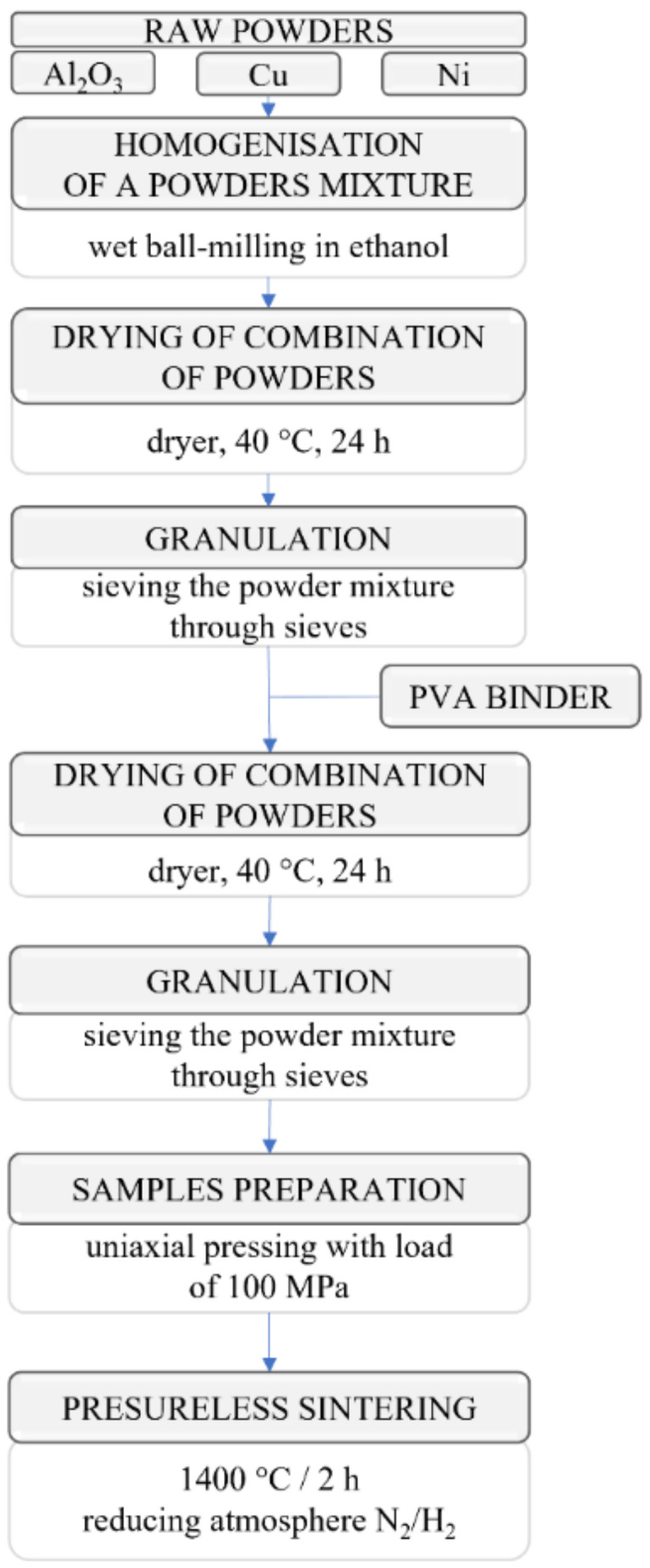
Scheme of the composite sample manufacturing processes.

Composite samples from the Al_2_O_3_-Cu-Ni system were produced in multiple stages, including uniaxial pressing and sintering. Figure 1 summarizes the individual stages of the process of producing composite moldings.

During the first step, the raw powders were homogenized in a Retsch (Retsch, Haan, Germany) PM400 planetary ball mill for 1 h at a speed of 300 rpm. The homogenization of the powders was carried out in ethanol. The container and balls used for the powder milling were corundum sinter. Next, the powder mass was dried in a laboratory dryer at 40 °C for 24 h until the alcohol was evaporated entirely. The powders were then sieved through sieves, starting with a 600 μm sieve, and ending with a 300 μm sieve, resulting in a homogeneous mixture of ceramic–metallic powders. In the next step, a binder consisting of a 10% polyvinyl alcohol (PVA) solution was added to the powder in an amount of 10% by weight based on the total weight of the powder and again sieved. Green body samples were produced from the prepared powder by uniaxial pressing. The applied load was equal to 100 MPa. The prepared samples were subjected to a free sintering process in the last step. The sintering was carried out in a Carbolite STF 16/75/450 furnace at a temperature of 1400 °C. The heating process was carried out as follows: heating 2 °C/min to 1400 °C, holding 2 h cooling 2 °C/min. Reduction in the atmosphere of 80% N_2_/20% H_2_ was used to limit the oxidation of the metallic components included in the obtained composites. Sintering conditions were selected in previous research [27]. The obtained samples were 20 mm in diameter and 5 mm high.

Three series of composite samples with different metallic phase content were produced: series I—5 vol.% of the metallic phase; series II—10 vol.% of metallic phase; series III—15 vol.% of metallic phase. In the obtained samples, the ratio of Cu to Ni was 1:1 in each series (series I—Cu:Ni—2.5 vol.%:2.5 vol.%; series II—Cu:Ni—5 vol.%:5 vol.%; series III—Cu:Ni—7.5 vol.%:7.5 vol.%).

### 2.3. Properties Characterization

The sintered samples were thoroughly examined using several research methods.

The selected physical properties of the compounds were determined using the Archimedes method. The density of the raw powders and sintered composites was measured by the Accu Pyc II 1340 helium pycnometer (AccuPyc 1340 II by Micrometrics, Norcross, GA, USA). The investigations were carried out in the two stages: 10 purges and 700 measurement cycles with 0.13 MPa fill pressure for both stages.

The observation using a digital light microscope Olympus LEXT OLS4100 (Olympus Scientific Solutions Americas (OSSA), Waltham, MA, USA) was conducted to obtain overview pictures of the samples produced. To reveal the microstructure of the raw powder and the obtained composites, scanning electron microscopy with energy-dispersive X-ray spectroscopy (EDS) was used (JEOL JSM-6610 scanning electron microscope, JEOL Ltd., Tokyo, Japan). The observations were made at an acceleration voltage of 15 kV. As part of the preparation of the metallographic specimen preparation, all composites used for the experiments were mounted in resin, ground with 80–3000 graduations abrasive papers, and then polished using 3 and 1 μm diamond suspensions. The prepared samples were coated with carbon using QUORUM Q150T ES sputter (QUORUM, Judges House, Lewes Road, Laughton, East Sussex., UNITED KINGDOM) to SEM observation.

The phase composition of the compounds was analyzed with X-ray diffraction (Rigaku Corporation, Tokyo, Japan), using a sealed copper tube X-ray source that produces Cu Kα radiation at a wavelength λ = 1.54178 Å. Running parameters were as follows: voltage 30 kV, current 15 mA, angular range 20° to 100°, step Δ2θ − 0.01°, counting time 1 s. The diffractometric data was processed via MDI JADE 7 software (Materials Data Inc., Livermore, CA, USA).

**Figure 2:**
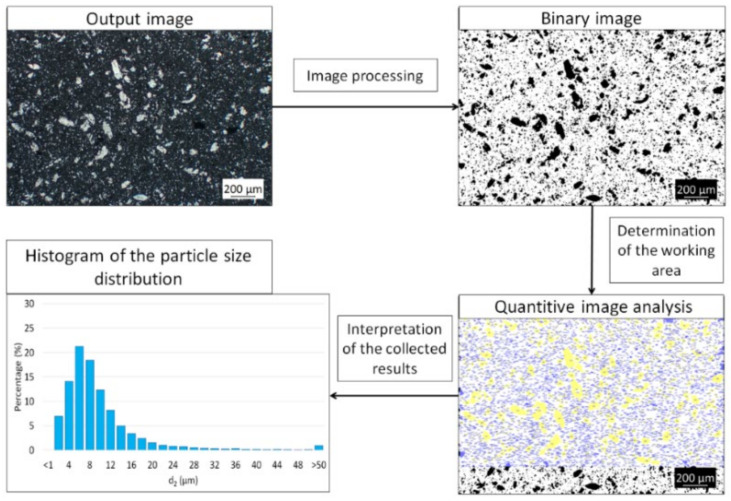
Diagram the image processing process to obtain the percentage share of the metallic phase.

Image analysis was performed using Micrometer computer software [28,29] on the SEM micrographs of the prepared cross-sections of the composites. Stereological analysis of images contained image processing, measurement, and explanation of received scores. Based on the average equivalent diameter of the metallic areas in the microstructure, determined by the software, the percentage share of different size fractions of the metallic particles was determined. The diagram of the image processing process to obtain the percentage share of the metallic phase is shown in Figure 2 The average values were calculated from the measurement of 20 pictures from different areas of the samples.

Magnetic measurements were carried out with a Lake Shore 7010 VSM magnetometer (Lake Shore Cryotronics, Inc., Westerville, OH, USA). The small piece of samples was placed on the magnetometer perpendicularly to the direction of the magnetic field. The measurements were made with the maximum intensity of the magnetic field: 2280 kA/m. The saturation magnetization was determined on the obtained magnetic hysteresis loops.

Hardness was determined using the Vickers hardness method. The measurements were carried out using a hardness tester HVS-30T hardness tester (Huatec Group Corporation, Beijing, China) on the surfaces prepared for metallographic observation. A load of 196 N and an applied load time of 10 s were used. The measurements were carried out on specimens subjected to metallographic preparation (grinding and polishing). The indents were made in randomly selected places on the sample. For each of the tested samples, 10 indents were made. The indentation method was used to determine the fracture toughness of the fabricated composites. Three equations, summarized in Table 2, were applied to establish the fracture toughness coefficient *K_IC_* value for comparative analysis.

Unified symbol designations have been implemented. For all equations applied, *E* stands for Young’s modulus, *HV* for Vickers hardness, *c* is the length of the crack from the center of the indentation to the crack tip, and *a* stands for half of the diagonal of the indentation.

## 3. Results and Discussion

### 3.1. Description of the Initial Powders

**Figure 3:**
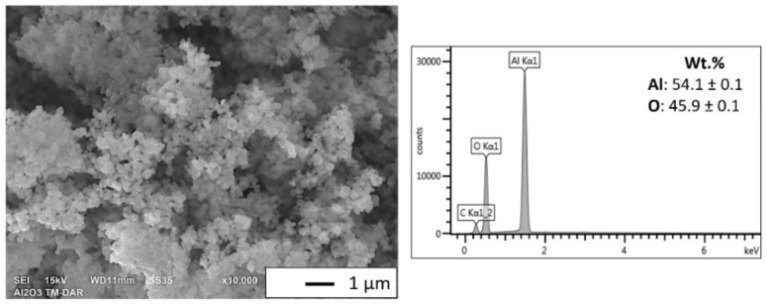
The morphology and EDS of Al_2_O_3_.

The morphology and EDX analysis results of Al_2_O_3_ are presented in Figure 3 SEM observations revealed that the Al_2_O_3_ particles were characterized by spherical morphology.

**Figure 4:**
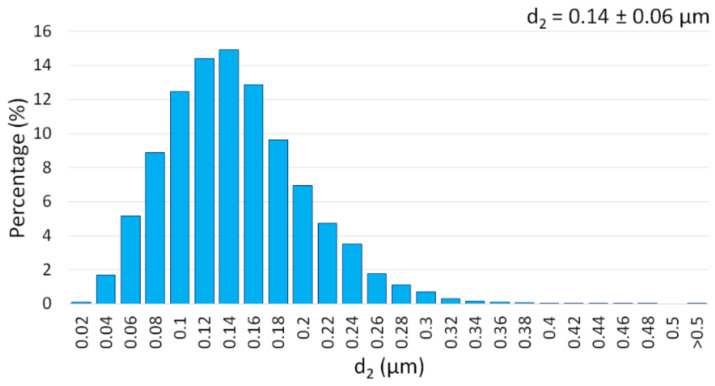
Histogram of the size of the Al_2_O_3_ powder.

The alumina particles were found to be firmly agglomerated. EDS analysis showed that Al_2_O_3_ powder contained 54.1 ± 0.1 wt.% aluminum and 45.9 ± 0.1 wt.% oxygen. Stereological analysis was also determined based on the size of Al_2_O_3_ powder calculated using SEM images. The results obtained are presented in a histogram in Figure 4.

The Al_2_O_3_ was characterized by the average particle size of 0.14 ± 0.06 μm. The obtained value of the actual powder size of the powder was similar to that quoted by the manufacturers (0.12 ± 0.02 µm). The density measured using a helium pycnometer for Al_2_O_3_ was 3.9782 g/cm^3^. The density value obtained was close to the density noted by the manufacturer (3.98 g/cm^3^).

**Figure 5:**
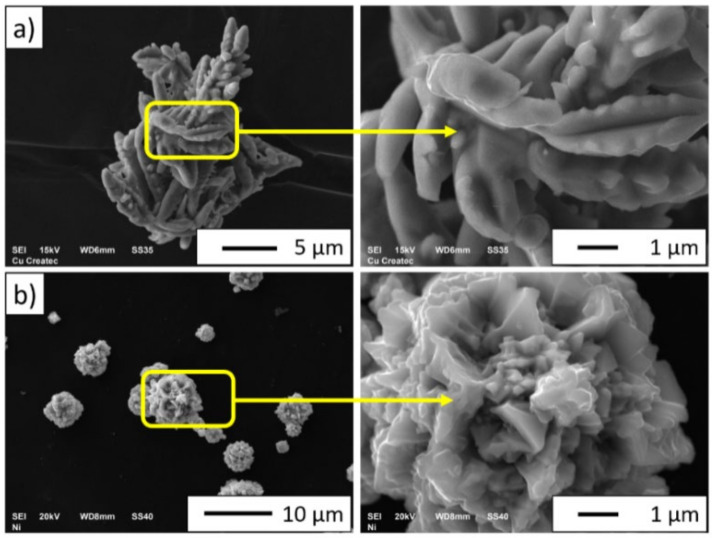
SEM images of the morphology of metal powders: (**a**) copper and (**b**) nickel.

Figure 5 shows the SEM images of the morphology of metallic powders: (a) copper and (b) nickel.

**Figure 6:**
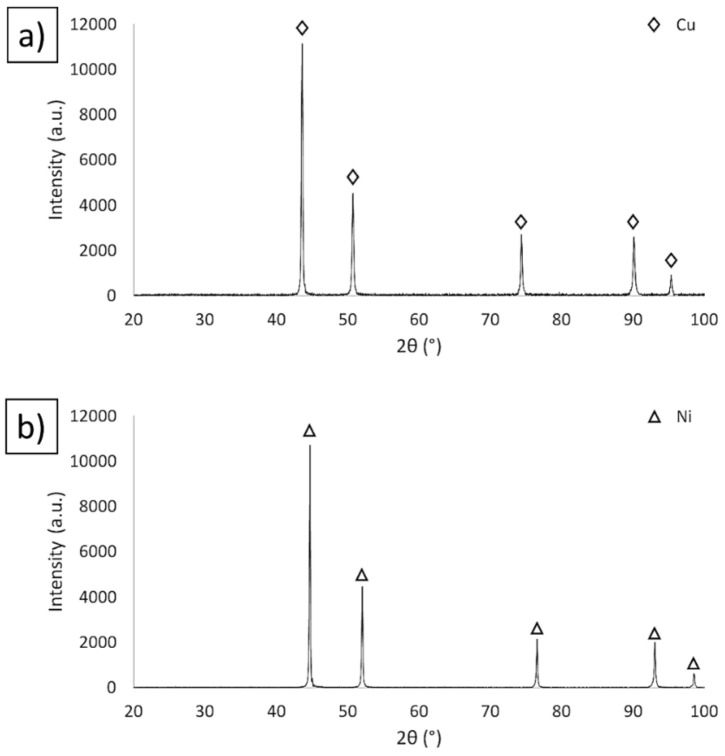
XRD of starting powders: (**a**) Cu and (**b**) Ni.

Analysis of the SEM images of the morphology showed that copper and nickel powders have irregular shapes. The dendritic shape of the copper powder (Figure 5a) suggests that it has been obtained by the electrolysis method. The nickel particles were found to exhibit cubic shapes (Figure 5b). The density measured using a helium pycnometer for Ni powder was 8.9102 g/cm^3^, while for Cu powder, it was 8.9613 g/cm^3^. The measured densities for the metallic powders were the same as those provided by the manufacturer (ρNi = 9.8 g/cm^3^, ρCu = 9.86 g/cm^3^). Figure 6 shows the results of the XRD investigation of the starting powders. The X-ray diffraction analysis revealed that the powders were single-phase: copper phase (JCPDS-PDF #98-000-0172) and cubic nickel phase (JCPDS-PDF#004-0850).

### 3.2. Description of the Composites

**Figure 7:**
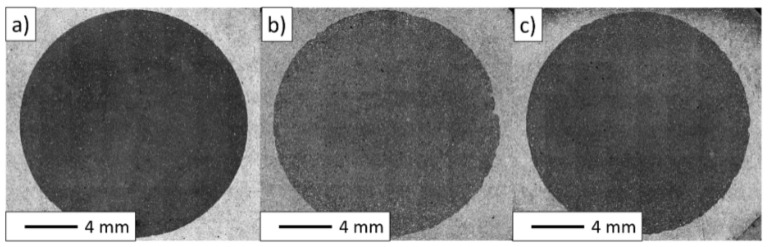
Observations carried out with the confocal microscope: (**a**) series I—5 vol.% of metallic phase; (**b**) series II—10 vol.% of metallic phase; (**c**) series III—15 vol.% of metallic phase.

Based on the overview observations performed via the digital light microscope (Figure 7), it can be observed that the composites from each series had a homogeneous microstructure, and the metallic phase was evenly allocated in the ceramic matrix. In the next section, the results of a stereological analysis are elucidated to confirm the distribution of metallic particles in composites. No visible defects in the structure characterized the samples obtained.

Table 3 summarizes the results of the selected physical properties of the obtained samples.

The analysis of the obtained results shows that, regardless of the content of the metallic phase in each series, the production method made it possible to obtain samples with a density exceeding 95% of the theoretical density. The highest relative density was characterized by series II, which contains 10% of the metallic phase (97.02 ± 0.31%).

In a previous study, the influence of temperature on composites from the Al_2_O_3_-Cu-Ni system was investigated. The samples were fabricated by single-axis pressing and sintering at three temperatures of 1100 °C, 1260 °C, and 1400 °C [27]. In the previous investigation, the highest density value characterized series sintered in the highest temperature examined. The relative density for Al_2_O_3_-Cu-Ni sintered at 1400 °C was equal to 96% of the theoretical value [27]. The obtained relative density value in this research is identical to that reported in previously published research results [27]. The differences in relative density for the two other series were close to each other and were within the error limits. A similar correlation was observed for open porosity and water absorption for series I and III samples.

The results obtained may indicate an optimal content of the metallic phase in the sample, which ensures good compaction of the composite for specific sintering conditions. However, this requires further research.

**Figure 8:**
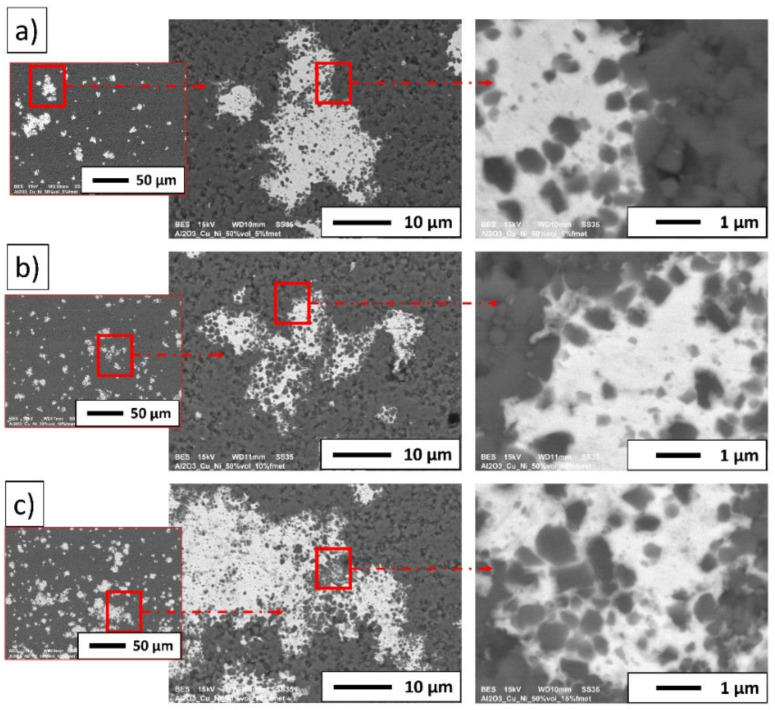
Typical microstructure for composites: (**a**) series I—5 vol.% of metallic phase; (**b**) series II—10 vol.% of metallic phase; (**c**) series III—15 vol.% of metallic phase.

Figure 8 shows the typical microstructure for the composites obtained.

Based on the obtained SEM images in backscattered electrons (BSE) mode, it was found that the light areas represent nickel/copper particles, while the dark areas represent alumina. Based on the microscopic observation, it was noted that the distribution of metallic particles in the matrix was not homogeneous, with a bit of tendency for aggregation. Metallic areas of various sizes were observed. SEM observation of the cross-section of the composite revealed a good connection between the metallic and ceramic phases. No delamination of the ceramic–metal interface was observed. However, SEM observation of the cross-section of the samples revealed the presence of single pores or voids in the structure. The presence of minor porosity in sintered composites may be due to the low wettability of the Al_2_O_3_ surface by the molten metal and the low melting temperature of copper. In the experiment, the sintering temperature of the composites (1400 °C) was higher than the Cu melting point (1085 °C). The alumina matrix had just begun the densification process; therefore, the specimens obtained could be porous. The molten copper did not wet the alumina surface due to the higher surface tension of the molten Cu, equal to the value 1240 mN/m at 1400 °C concerning the surface energy alumina (sapphire), which was equal to the value 700 mJ/m^2^ at 2080 °C [24,33,34]. Commonly, this promotes the leakage of liquid copper through the open pores. Previous research shows that the addition of nickel can limit the flow of liquid copper during sintering [24,33,34]. When molten copper comes in contact with solid nickel during the sintering process, a CuNi solution may be formed. The literature shows that depending on the copper-to-nickel ratio, the melting temperature of the copper–nickel alloy is between 1170 °C and 1240 °C [24,33,34]. The higher melting point of CuNi gives time for closing the open porosity of the alumina matrix, resulting in the densification of the sinters. In connection with this in prepared samples, the leakage of molten copper was not observed. The molten copper flowed through the alumina matrix pores, and the low wettability of ceramics by the molten metal during the sintering process and the possible formation of CuNi phases decreased densification, leading to some porosity in final composites [24,33,34].

**Figure 9:**
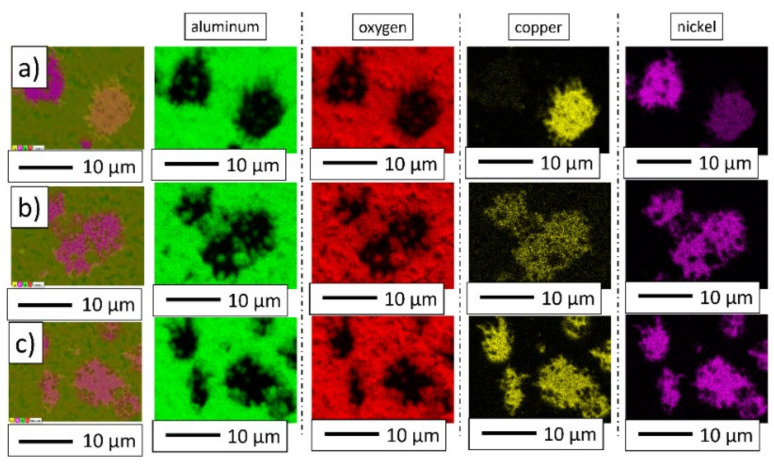
Distribution of elements on the surface of composites: (**a**) series I—5 vol.% of metallic phase; (**b**) series II—10 vol.% of metallic phase; (**c**) series III—15 vol.% of metallic phase.

Energy-dispersive X-ray spectroscopy was performed to indicate the distribution of individual elements in the produced composites. Figure 9 shows the distribution of the elements on the surface of composites.

The maps of the elements distribution on the samples’ surfaces revealed the presence and the concentrations of aluminum, oxygen, copper, and nickel. The observation indicated that the concentration of aluminum and oxygen corresponded to the composite matrix. The samples were found to contain areas rich in nickel and copper simultaneously. Most likely, these may be the regions corresponding to the CuNi solid solution. Unfortunately, energy-dispersive X-ray spectroscopy measurements did not estimate whether the nickel and cooper areas corresponded to a solid solution. X-ray diffraction (XRD) measurements were performed to determine the phase composition in samples.

**Figure 10:**
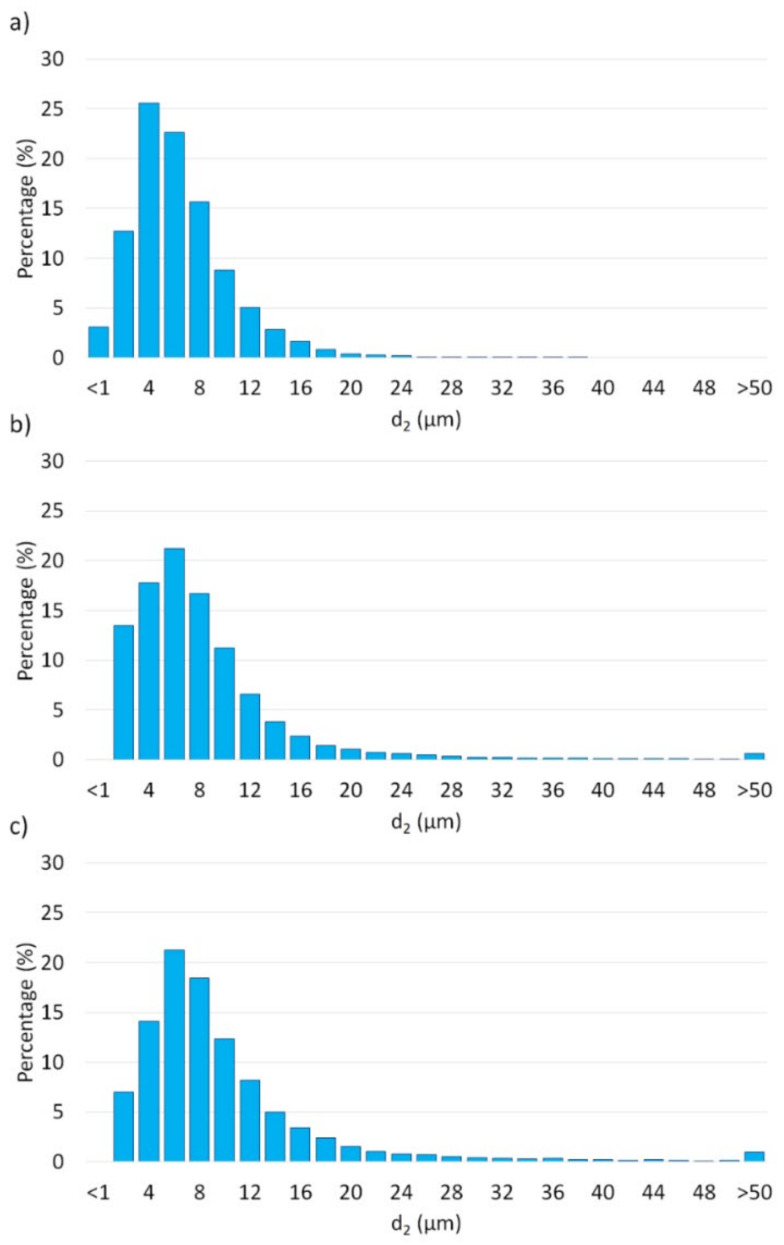
Histograms exemplify the size distribution of metal phases in composites: (**a**) series I—5 vol.% of metallic phase; (**b**) series II—10 vol.% of metallic phase; (**c**) series III—15 vol.% of metallic phase.

Figure 10 shows histograms of the metallic phase size distribution of the metallic phase in the tested samples.

Analysis showed that all obtained histograms had unimodal distributions. The analysis revealed that, along with the increasing amount of metallic phase in samples, the percentage amount of the larger particles in the microstructure also increased. Our investigation indicated that series I was characterized by the ceramic matrix’s smallest average metallic phase size. The samples containing 5 vol.% of metallic particles had an average metallic phase size range from 0.77 to 37.47 μm. For series II, the metallic phase ranged from 1.69 to 144.92 μm. In comparison, the samples of series III characterized metallic phase sizes ranging from 1.55 to 159.26 μm.

**Figure 11:**
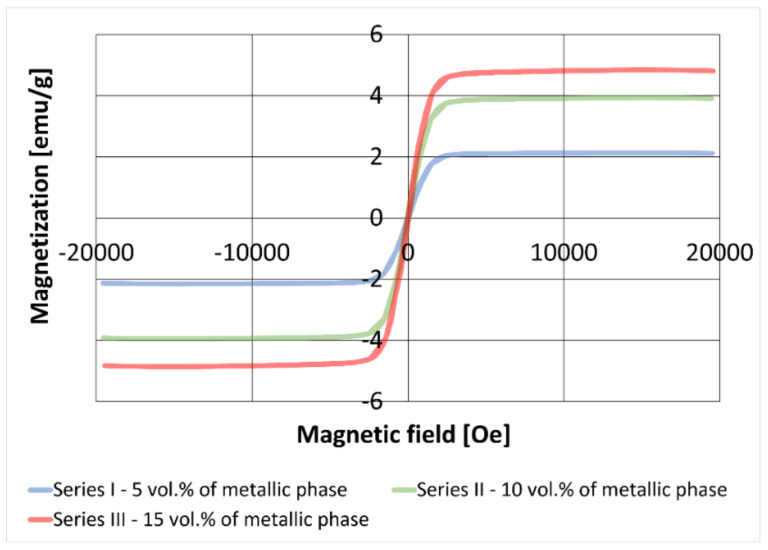
Hysteresis loops of prepared composites.

The dependence of magnetization on the magnetic field strength was recorded for all composites tested. Figure 11 shows an example of hysteresis loops for samples with different content contributions of the metallic phase, sintered at a temperature of 1400 °C. The results showed that the obtained composites exhibited ferromagnetic properties due to nickel and nickel-rich phases. It was found that the saturation of magnetization of composites increased with an increase in the proportion of the metallic phase.

**Figure 12:**
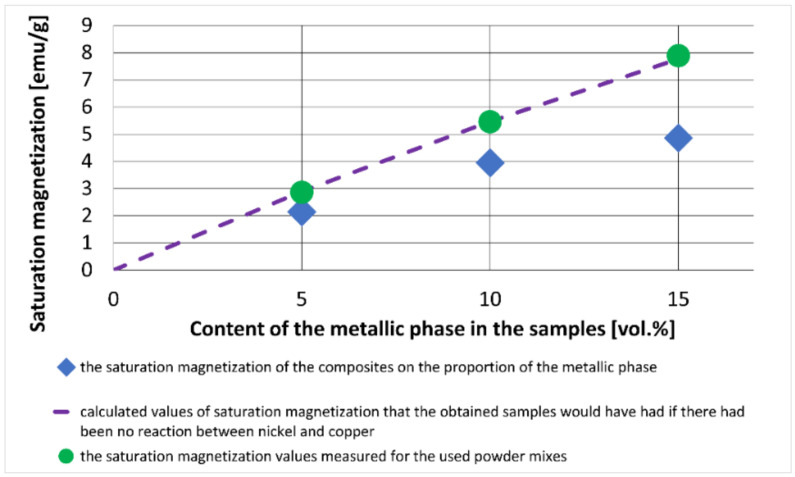
Results of measurements of saturation magnetization.

Figure 12 shows the dependence of the saturation magnetization of the obtained composites on the proportion of the metallic phase.

The saturation magnetization (Ms) is the maximum magnetic moment per unit volume for a magnetic material. A line is also marked on the graph, showing the calculated values of saturation magnetization that the obtained samples would have had if there had been no reaction between nickel and copper. The calculations were made assuming that the measured value of saturation magnetization of the nickel used was 54.715 emu/g. These results agreed with the saturation magnetization values measured for the used powder mixes.

Data from the literature show that solutions with Ni content greater than 77% by weight should have ferromagnetic properties at room temperature [35]. The addition of copper reduces the saturation magnetization of CuNi solutions, which was described, among others, in the publication [36,37] for Cu-Ni nanoparticles. Therefore, the reaction between the nickel and copper particles should be expected to decrease the magnetization of the composite.

The results revealed that all tested composites had lower saturation magnetization than those recorded for the raw powders. A subsequent decrease in this property is probably dependent on the degree of reaction between the metallic component particles. The reaction between the copper and nickel particles in the Al_2_O_3_ matrix may occur only in their common contact areas. Microscopic observations showed that some of the nickel particles remained isolated from the copper, so it was impossible to lose the ferromagnetic properties of the composite, i.e., ultimately obtaining a solution containing 50% of each of these elements in the entire volume. Therefore, changes in saturation magnetization, compared with the raw powder mixtures, were more significant with an increasing proportion of the metallic phase, as the probability of contact between the particles of both metals increased.

**Figure 13:**
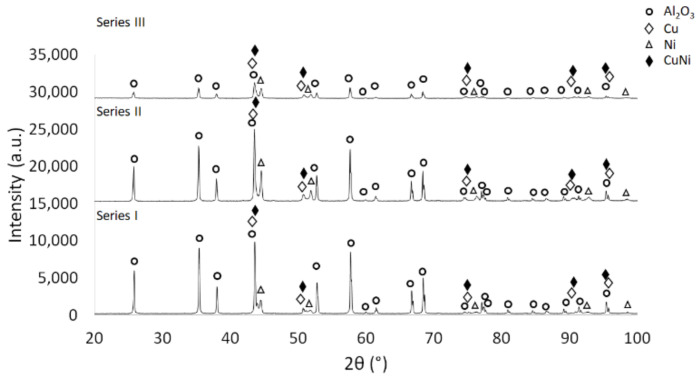
XRD diffractograms for obtained composites with different content contributions of metallic phase after sintering.

The next step focused on a phase analysis of the sintered specimens. The phase composition of the sintered samples is shown in Figure 13 The obtained results indicated the presence of four phases in all investigated series: Al_2_O_3_, Cu, Ni, and CuNi solid solution. The research demonstrated that the intensity of the individual phases, depending on the composition of composites, was different.

**Figure 14:**
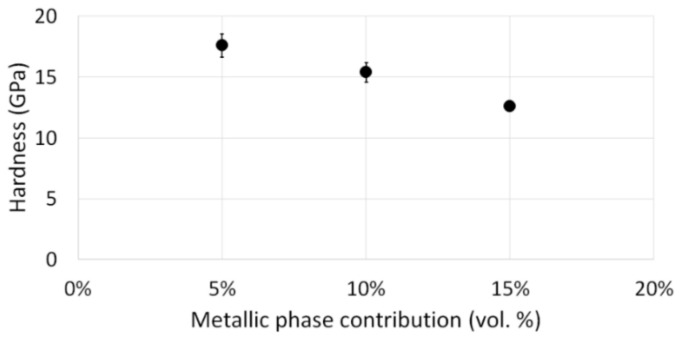
Vickers hardness results.

The Vickers hardness results achieved for the individual series are presented in Figure 14.

Examining the obtained results, the correlation between the content of the metallic phase in the composite and its hardness was determined. As the metallic phase content in the composite structure increased, a decrease in its hardness was observed. Therefore, the material with the lowest 5 vol.% content of the metallic phase showed the highest hardness, reaching a hardness equal to 17.60 ± 0.96 GPa. The composite with the highest metallic phase content of 15 vol.% was characterized by the lowest hardness, equal to 12.6 ± 0.36 GPa.

**Figure 15:**
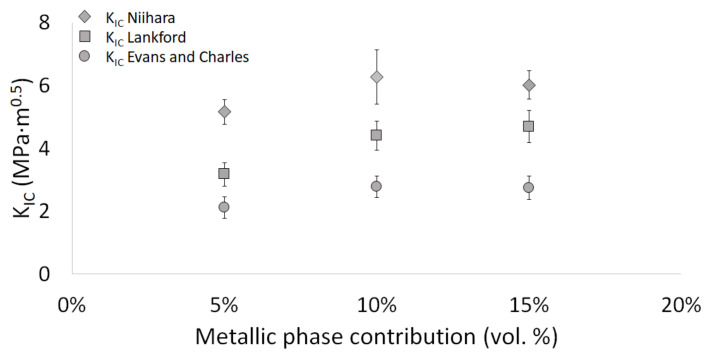
Fracture toughness of the sintered composites with different metal phase content contribution.

Examination of the obtained fracture toughness values, presented in Figure 15, demonstrated a strong correlation between the obtained *K_IC_* values and the formula applied for its determination. Accordingly, the Niihara equation provided the highest fracture toughness values, ranging between 5.16 and 6.27 MPa·m^0.5^. In contrast, the Evans and Charles equation gave the lowest values of the *K_IC_* coefficient, within the range of 2.11–2.78 MPa·m^0.5^. Regardless of the selected equation, the fracture toughness values among the series with different metallic phase content remained similar.

However, despite some subtle differences, the analysis of the achieved results revealed that with the increase in the metallic phase content, hence phases with higher ductility, the material’s fracture toughness improved in the ceramic matrix. Therefore, regardless of the equation applied, this study’s lowest fracture toughness values were observed in the composite with 5 vol.% metallic phase content. The *K_IC_* values for this series amounted to 5.16 ± 0.39 MPa·m^0.5^ for the Niihara equation, 3.17 ± 0.37 MPa·m^0.5^ for the Lankford equation, and 2.11 ± 0.27 MPa·m^0.5^ applying Evans and Charles equation for the calculations. The increase in metallic phase volume resulted in improved fracture toughness properties of the material. However, the differences in *K_IC_* values observed for the series with 10 vol.% and 15 vol.% of the metallic phase content were only minor. In the Lankford equation case, the highest fracture toughness value was recorded for a sample with 15 vol.% metallic phases, with *K_IC_* equal to 4.69 ± 0.51 MPa·m^0.5^. In contrast, for the Niihara and Evans and Charles equations, the maximum values were characterized by the series with 10 vol.% metallic phase with *K_IC_* amounting to 6.27 ± 0.87 MPa·m^0.5^ (Niihara equation) and 2.78 ± 0.27 MPa·m^0.5^ (Evans and Charles eq.), respectively. The fracture toughness values achieved for each series are summarized in Table 4.

Unfortunately, scarce research is available in the literature that directly addresses the Al_2_O_3_-Cu-Ni ceramic–metal composite system. However, the results we obtained stand in good correlation with the previous studies conducted by the team on this system. In the work of Zygmuntowicz et al., hardness equal to 11.04 GPa was achieved for samples of the Al_2_O_3_-Cu-Ni system with 15 vol.% metallic phase content obtained by slip casting. A *K_IC_* value also characterized these samples from the Niihara equation equal to 6.17 MPa·m^0.5^. Comparatively, the hardness obtained in the same study for the pure Al_2_O_3_ sample exceeded that of the composite and reached 16.03 GPa, while the fracture toughness was found to be lower and equal to 4.97 MPa·m^0.5^ [35]. Applying uniaxial pressing and the PPS method to produce Al_2_O_3_-Cu-Ni specimens with 15 vol.% metallic phase content resulted in samples with slightly lower hardness, 9.38 GPa and 7.69 GPa for PPS and pressed samples, respectively, but with very similar fracture toughness values. The *K_IC_* values achieved for the PPS and pressed samples amounted to 6.03 MPa·m^0.5^ and 5.62 MPa·m^0.5^, respectively [15]. Previous studies conducted on uniaxially pressed samples from Al_2_O_3_-Cu and Al_2_O_3_-Cu-Ni systems showed that the addition of Ni to the metallic phase, although slightly reducing the *K_IC_* value, had a beneficial effect on the mechanical properties of the material. The hardness obtained for these samples, characterized by a 10 vol.% metallic phase, was equal to 9.28 GPa for the sample with copper and 15.40 GPa for the sample with copper and nickel. *K_IC_* values were comparable, ranging between 6.27 and 6.45 MPa·m^0.5^ [27].

Although there are few existing reports in the literature regarding the Al_2_O_3_-Cu-Ni ternary system, both Al_2_O_3_-Cu and Al_2_O_3_-Ni composites are well represented. The addition of copper and nickel to the ceramic matrix improves the material’s fracture toughness compared to the pure matrix. The *K_IC_* values, determined from Miyoshi’s equation, for both systems with 10 vol.% metallic phase content were similar, ranging between 4.5 and 4.8 MPa·m^0.5^ [38]. Likewise, for the Al_2_O_3_-Ni composites produced by the SPS method, a reduction in hardness was observed with the increase in the weight content of nickel in the structure, along with an enhancement of the fracture toughness. The fracture toughness in this study, determined from the Evans and Charles equation, was in the range of 3.89–3.97 MPa·m^0.5^, depending on the metallic phase content [39].

Regarding Al_2_O_3_-Cu composites fabricated by uniaxial pressing, the change in the *K_IC_* value with increasing copper content in the structure was more noticeable. The *K_IC_* values determined from the same equation ranged from 3.59 to 7.44 MPa·m^0.5^ [40] for similar metal weight contents. It should be noted that both fracture toughness and hardness depend on various factors, such as the manufacturing method and the initial powders involved, hence the variations in values observed to some extent.

## 4. Conclusions

Ceramic–metal composites based on Al_2_O_3_, Ni, and Cu were prepared by uniaxial pressing. Three series of composite with different metallic phase content (5 vol.%, 10 vol.%, 15 vol.%) were fabricated. In this study, Cu was partially substituted by Ni. The addition of Ni powder was favorable in view of the fact that Ni particles facilitated the spread of liquid copper during sintering in samples. The microscopic observation showed that the uniaxial pressing technique allowed obtaining no homogenous samples. Metallic areas of various sizes were noticed. It was found that regardless of the content of the metallic phase in each series, the obtained composites had a relative density exceeding 95%. The stereological analysis indicated that, along with the increasing amount of metal phase in composites, the percentage amount of the larger particles in microstructure also increased. The XRD analysis indicated the presence of four phases in all studied series—Al_2_O_3_, Cu, Ni, and Cu-Ni solid solution. The magnetic measurements exhibited that the fabricated composites had ferromagnetic properties due to nickel and nickel-rich phases. The results revealed that all tested composites had lower saturation magnetization than those recorded for the started powders. This is due to the fact that solutions with Ni content greater than 77% by weight were characterized by ferromagnetic properties at room temperature. The addition of copper reduced the saturation magnetization of CuNi solutions. Consequently, the reaction between the Ni and Cu particles decreased the magnetization of the specimens. Based on hardness measurements, it was found that the hardness of the samples decreased with the increase in the metallic phase content. The samples containing 5 vol.% metallic phase had a hardness equal to 17.60 ± 0.96 GPa, while the samples with 10 vol.% metallic phase had a hardness equal to 15.40 ± 0.81 GPa, and the samples with 15 vol.% metallic phase had a hardness equal to 12.6 ± 0.36 GPa. The trials presented in this manuscript are preliminary studies. Investigations on the other mechanical properties of Al_2_O_3_-Cu-Ni composites are in progress, and the results will be published in subsequent articles.

## Figures and Tables

**Table 1:** Properties of the base materials.

	Al_2_O_3_	Cu	Ni
Purity (%)	99.9	99.8	99.8
Density (g/cm^3^)	3.98	8.96	8.9
Mean Particle Size (μm)	0.12 ± 0.02	<150	3–7

**Table 2:** Equations applied to the *K_IC_* determination.

Author	Equation	
Niihara	$K_{IC}=0.018\cdot HV^{0.6}\cdot E^{0.4}\cdot {\left(l\right)}^{-0.5}\cdot 0.5d$	[30]
Evans and Charles	$K_{IC}=0.16\cdot \left(HV\cdot a^{0.5}\right)\cdot {\left(\frac{c}{a}\right)}^{-1.5}$	[31]
Lankford	$K_{IC}=0.0782\cdot \left(HV\cdot a^{0.5}\right)\cdot {\left(\frac{E}{HV}\right)}^{0.4}\cdot {\left(\frac{c}{a}\right)}^{-1.56}$	[32]

**Table 3:** Selected physical properties of the obtained samples.

	Series I—5 vol.% of the Metallic Phase	Series II—10 vol.% of the Metallic Phase	Series III—15 vol.% of the Metallic Phase
Theoretical density (g/cm^3^)	4.237	4.484	4.731
Relative density (%)	96.07 ± 0.37	97.02 ± 0.31	96.06 ± 0.78
Open porosity (%)	1.23 ± 0.37	0.08 ± 0.04	1.60 ± 0.61
Water absorption (%)	0.30 ± 0.09	0.02 ± 0.01	0.35 ± 0.14

**Table 4:** Fracture toughness of the sintered composites with different metallic phase content contributions.

	Series I—5 vol.% of the Metallic Phase	Series II—10 vol.% of the Metallic Phase	Series III—15 vol.% of the Metallic Phase
Niihara	5.16 ± 0.39	6.27 ± 0.87	6.01 ± 0.45
Lankford	3.17 ± 0.37	4.41 ± 0.46	4.69 ± 0.51
Evans and Charles	2.11 ± 0.27	2.78 ± 0.27	2.74 ± 0.29

## Data Availability

Data sharing is not available.
